# Tumor-associated microbiome features of metastatic colorectal cancer and clinical implications

**DOI:** 10.3389/fonc.2023.1310054

**Published:** 2024-01-18

**Authors:** Ho Jung An, Mira A. Partha, HoJoon Lee, Billy T. Lau, Dmitri S. Pavlichin, Alison Almeda, Anna C. Hooker, Giwon Shin, Hanlee P. Ji

**Affiliations:** ^1^Department of Medical Oncology, St. Vincent’s Hospital, College of Medicine, The Catholic University of Korea, Seoul, Republic of Korea; ^2^Division of Oncology, Department of Medicine, Stanford University School of Medicine, Palo Alto, CA, United States; ^3^Department of Electrical Engineering, Stanford University, Palo Alto, CA, United States

**Keywords:** tissue microbiome, metastasis, colon, rectum, adenocarcinoma

## Abstract

**Background:**

Colon microbiome composition contributes to the pathogenesis of colorectal cancer (CRC) and prognosis. We analyzed 16S rRNA sequencing data from tumor samples of patients with metastatic CRC and determined the clinical implications.

**Materials and methods:**

We enrolled 133 patients with metastatic CRC at St. Vincent Hospital in Korea. The V3-V4 regions of the 16S rRNA gene from the tumor DNA were amplified, sequenced on an Illumina MiSeq, and analyzed using the DADA2 package.

**Results:**

After excluding samples that retained <5% of the total reads after merging, 120 samples were analyzed. The median age of patients was 63 years (range, 34–82 years), and 76 patients (63.3%) were male. The primary cancer sites were the right colon (27.5%), left colon (30.8%), and rectum (41.7%). All subjects received 5-fluouracil-based systemic chemotherapy. After removing genera with <1% of the total reads in each patient, 523 genera were identified. Rectal origin, high CEA level (≥10 ng/mL), and presence of lung metastasis showed higher richness. Survival analysis revealed that the presence of *Prevotella* (*p* = 0.052), *Fusobacterium* (*p* = 0.002), *Selenomonas* (*p*<0.001), *Fretibacterium* (*p* = 0.001), *Porphyromonas* (*p* = 0.007), *Peptostreptococcus* (*p* = 0.002), and *Leptotrichia* (*p* = 0.003) were associated with short overall survival (OS, <24 months), while the presence of *Sphingomonas* was associated with long OS (*p* = 0.070). From the multivariate analysis, the presence of *Selenomonas* (hazard ratio [HR], 6.35; 95% confidence interval [CI], 2.38–16.97; *p*<0.001) was associated with poor prognosis along with high CEA level.

**Conclusion:**

Tumor microbiome features may be useful prognostic biomarkers for metastatic CRC.

## 1 Introduction

The human microbiome plays a fundamental role in maintaining physiological homeostasis. Microbial dysbiosis contributes to the pathophysiology of human diseases, including colorectal cancer (CRC), which has a close physical proximity with the diverse microbial populations in the colon (1, 2). The tumor-associated microbiome plays an important role in colorectal cancer biology given its direct contact with the local tumor microenvironment (TME). DNA sequencing analysis conducted directly on CRC tumors provides insights into the cancer-associated microbiota that is in direct contact with the local TME of CRCs. As an example, many studies investigating the tumor microbiome of CRC have demonstrated an enrichment of *Fusobacterium nucleatum* (*F. nucleatum*) in colon carcinogenesis (3–5).

Other studies have delineated how specific microbiome may impact CRC pathophysiology. Preclinical studies have shown that *Bacteroides fragilis* and *Escherichia coli* secrete enterotoxins that induce pro-inflammatory immune responses and DNA damage. The direct adhesion of *F. nucleatum* to colon cells has been shown to exert similar effects. There is mounting evidence supporting the hypothesis that these effects promote colon carcinogenesis (6–10). Identification of specific microbiome alterations may provide clinically relevant information when screening for early-stage CRC (11–16).

Specific microbiome features are also associated with CRC prognosis. For example, the presence of *F. nucleatum* in CRC tissues is correlated with advanced stage, microsatellite instability, and poor prognosis in patients with CRC who have undergone surgical resection (17, 18). Microbiome composition also influences the efficacy of cancer immunotherapy (19–21) and chemotherapy via various mechanisms (22–26).

Most microbiome studies on CRC have been conducted using tumor samples from surgical resections, which are typically performed with curative intent in lower-stage (i.e., I–III) patients. Therefore, little is known about the clinical implications of the microbiome composition in patients with stage IV metastatic CRC receiving systemic treatment. In this study, we conducted microbiome 16S rRNA sequencing using tumor tissues from patients with initially stage IV CRC and determined the correlation between the tissue microbiome and clinical outcomes.

## 2 Materials and methods

### 2.1 Study population

All patients had a histological diagnosis of colorectal adenocarcinoma, initially as a stage IV disease, and received 5-fluouracil (5-FU)-based systemic chemotherapy and/or biological agents (cetuximab or bevacizumab) after tissue sampling. Treatment response was determined using the treatment evaluation criteria for solid tumors (v. 1.1) (27).

This study was approved by the Institutional Review boards (IRB) of the St. Vincent’s Hospital (No. VC19TESI0114), and Stanford Hospital (IRB number 55131). All the patients provided written informed consent to participate in this study. This study was conducted in accordance with the principles of the Declaration of Helsinki.

### 2.2 Sequencing analysis

The original sample set comprised 133 CRC tissue samples biopsied from patients with stage IV disease. Two scrolls of 7 to 8 µm sections were sliced from the formalin-fixed, paraffin-embedded (FFPE) block and stored at –20°C until their processing. Total genomic DNA was extracted using Maxwell® 16 FFPE Tissue LEV DNA Purification Kit from Promega (Madison, Wisconsin, USA). ZymoBIOMIC Microbial Community DNA Standard (Zymo Research, Catalog No. D6300) was used as a positive control, and DNA-free water was used as a negative control for 16S sequencing and data analysis. For 16S rRNA gene amplicon library preparation, we used 16S metagenomic sequencing library preparation according to the manufacturer’s protocol (Illumina, San Diego, California, USA). Briefly, 200 ng of mucosal DNA was amplified using primers targeting the V3-V4 variable region of the 16S rRNA gene.

16S forward primer:

5’-TCGTCGGCAGCGTCAGATGTGTATAAGAGACAGCCTACG GGNGGCWGCAG-3’;

16S reverse primer:

5’-GTCTCGTGGGCTCG GAGATGTGTAT AAGAGACAGGACTACHVGGGTATCTAATCC-3”.

The DNA sample was adjusted to a final concentration of 12 pM, subjected to PCR amplification and sequence library processing, and sequenced on an Illumina MiSeq platform (Illumina Technologies, USA).

### 2.3 16S rRNA data analysis

Cutadapt software (version 1.14,-m30) was used to remove the primer and adapter sequences. After filtering low-quality reads and merging, 13 samples had low number of reads and were excluded from the study. This step left 120 samples for further downstream analysis.

The Divisive Amplicon Denoising Algorithm 2 (DADA2) program was used to analyze the data and generate amplicon sequence variant (ASV) tables (**Supplementary Figure S1**). Then, we converted these tables into phyloseq objects using the “phyloseq” package (v. 1.24.2). Features with ambiguous genus annotations were discarded, and ASVs found in at least 1% of each sample were selected. Microbiomes detected in the negative control were subtracted from the in silico dataset.

Two alpha indices were chosen to depict taxon diversity between the groups: richness determined by number of Observed-ASVs per sample (observed ASV) and evenness determined by diversity of ASVs per sample (Shannon index). The beta diversity to depict intergroup dissimilarities was measured using UniFrac distances and visualized using principal component analysis (PCA). Linear discriminant analysis with effect size (LEfSe) algorithm was used to define differential microbiome patterns (28). The relative microbiome abundance between the groups was visualized using a heat map.

### 2.4 Statistical analysis

We performed a chi-squared test, Wilcoxon signed-rank test, and Spearman’s correlation to evaluate the results among the different pipelines. The Mann–Whitney *U* test or one-way ANOVA test was used for alpha diversity comparison. Permutational multivariate analysis of variance (PERMANOVA) was used to compare the beta diversity.

Progression-free survival (PFS) was measured from the date of first-line systemic chemotherapy to the date of progression or censored at the last follow-up date. Overall survival (OS) was measured from the date of first-line systemic chemotherapy until death from any cause, or the last censored date during follow-up. PFS and OS were calculated using the Kaplan–Meier method, and differences in survival between groups were compared using the log-rank test. Variables with *p* value <0.07 were included in the Cox regression multivariable model, and adjusted hazard ratios (HR) with 95% confidence intervals (CI) were calculated. A *p* value <0.05 was considered statistically significant. Statistical analyses were performed using R software (version 3.6.1).

## 3 Results

### 3.1 Patient characteristics

The baseline characteristics of the 120 enrolled patients are summarized in **Table 1**. The median age was 63 years (range, 34–82 years), and 76 patients (63.3%) were male. All patients were of Korean ethnicity. The distribution of the primary tumor sites was 27.5% in the right colon, 30.8% in the left colon (excluding the rectum), and 41.7% in the rectum. Twenty-one (18.5%) patients showed an involvement of three metastatic organs or more, which was defined as a high tumor burden.

**Table 1:** Baseline characteristics of the study population.

Characteristics		No. of patients N = 120 (%)
Age	Median (range)	63 (34–82)
Gender	Male	76 (63.3)
	Female	44 (36.7)
Ethnicity	Korean	120 (100%)
ECOG performance status	0/1	86 (71.7)
	≥2	34 (28.3)
Stage	IV	120 (100.0)
Primary tumor location	Right side colon	33 (27.5)
	Left side colon	37 (30.8)
	rectum	50 (41.7)
Tumor histology	Adenocarcinoma	120 (100.0)
Tumor differentiation	Well	12 (10.0)
	Moderate	100 (83.3)
	Poor	8 (6.7)
CEA (ng/mL)	Median (range)	8 (0.27–>1,500)
Number of organ metastasis	1	56 (46.7)
	2	43 (35.8)
	≥3	21 (18.5)
Site of organ metastasis	Lung	30 (25.0)
	Liver	55 (45.8)
	Lymph node	28 (23.3)
	Peritoneum	28 (23.3)
KRAS/NRAS mutation	Yes	62 (51.7)
Microsatellite instability	High	2 (1.7)
1^st^ line cytotoxic agent	FOLFOX	35 (29.2)
	FOLFIRI	85 (70.8)
1^st^ line biologic agent	Cetuximab	47 (39.2)
	Avastin	66 (55.0)
	None	7 (5.8)
Overall response rate^*^	Complete or partial response	51 (42.5)
Progression free survival	Median (range)	8.9 months (1.0–52.0)
Overall survival	Median (range)	18.1 months (3.6–59.4)

ECOG, Eastern Cooperative Oncology Group; CEA, Carcinoembryonic antigen; FOLFOX, 5-FU,/oxaliplatin/leucovorin; FOLFIRI, 5-FU/irinotecan/leucovorin.

^*^1^st^ line chemotherapy.

During the median follow-up period of 17.9 months (1.0–59.0), 51 (42.5%) patients showed responses (complete or partial responses), and the median PFS was 8.9 months (1.0–52.0) after first-line treatment. Twenty-nine (24.2%) patients survived more than 24 months, and the median OS was 18.1 months (3.6–59.4).

### 3.2 Microbiome composition

In total, 13.3 million reads were generated from 120 CRC tissues. The median read length was approximately 392 bp (range, 283–590 bp). A total of 43,551 ASVs were assigned based on 97% similarity; however, 30,693 (70.5%) remained unclassified at the genus level. We removed unclassified ASV, and filtered out genera that showed an abundance of less than 1% in each patient to remove possible artifacts of sequencing errors or clustering algorithms. Finally, 523 genera were identified and analyzed. *Pseudomonas* was the most abundant, followed by *Clostridium, Bacteroides, Rombroutsia, Flectobacillus, and Staphylococcus*, in descending order (**Supplementary Figure S2**).

### 3.3 Association of alpha diversity and clinical parameters

**Figure 1:**
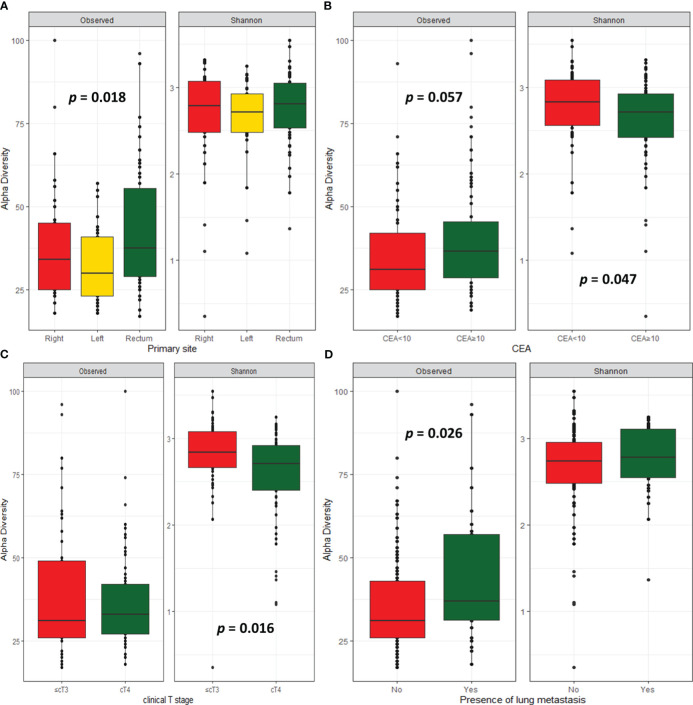
Microbial diversity. Alpha-diversity index according to primary site **(A)**, CEA level **(B)**, clinical T stage **(C)**, and presence of lung metastasis **(D)**.

Alpha diversity describes the microbial diversity in terms of richness (observed ASVs) and evenness (Shannon index). Alpha diversity analysis revealed that cancer tissues originating from the rectum showed higher microbiome richness than those from left- or right-sided CRC (*p* = 0.018), but there was no difference in microbiome diversity (**Figure 1A**). A trend towards higher microbiome richness (*p* = 0.057), but lower diversity (*p* = 0.047) was observed in CRC tissues with high serum CEA level (≥ 10 ng/mL, **Figure 1B**). CRC tissues with peritoneal invasion (cT4) showed lower microbiome diversity than those without peritoneal invasion (*p* = 0.016, **Figure 1C**). Patients with lung metastasis showed higher microbiome richness than those without lung metastasis (*p* = 0.026; **Figure 1D**). There were no significant differences in alpha diversity according to clinical outcomes, including response rate, progression, and survival.

### 3.4 Association of beta diversity and clinical parameters

**Figure 2:**
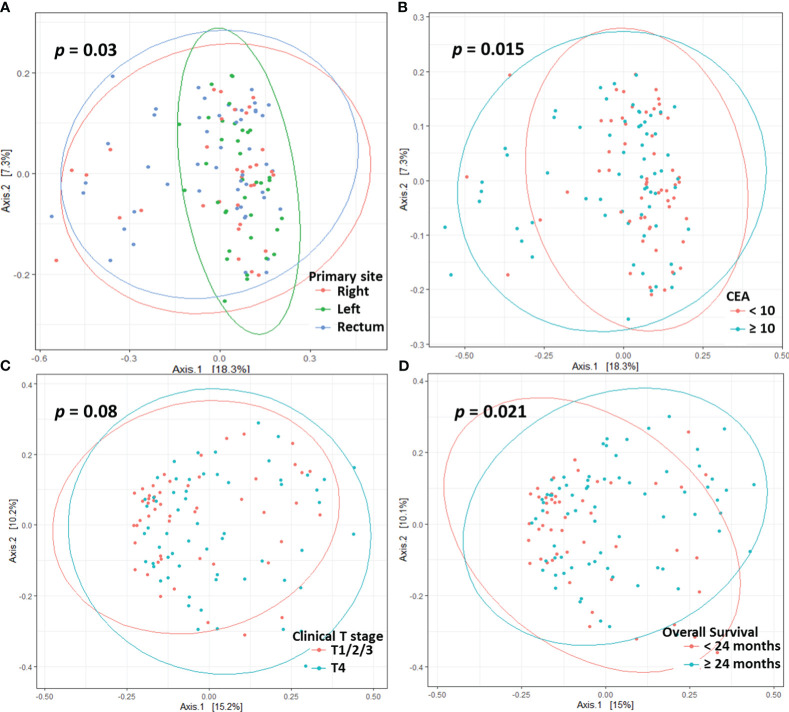
Principle co-ordinates analysis based on the Bray-Curtis distance according to primary site **(A)**, CEA level **(B)**, clinical T stage **(C)**, and overall survival **(D)**.

**Figure 3:**
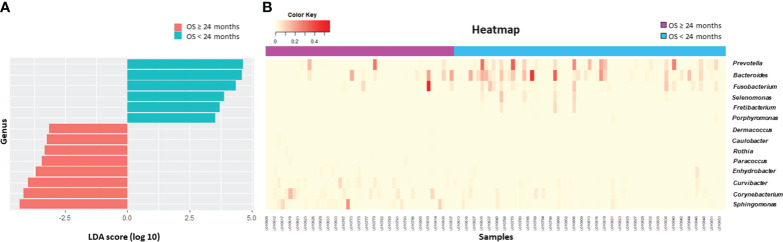
Survival analysis. Differential microbiome abundance between short term (<24 months) and long term (≥24 months) survivors analyzed by linear discriminate analysis with effect size measurement **(A)**. The listed genera were significantly gathered for their respective groups (*p*<0.05). Heatmap represents the relative abundance of each genera between two groups **(B)**.

Beta diversity differed according to primary site (*p* = 0.03, **Figure 2A**), serum CEA level (*p* = 0.015, **Figure 2B**), or clinical T stage (*p* = 0.08, **Figure 2C**). Patients who survived longer than 24 months (long-term survivors) showed distinct beta diversity compared to those who died within 24 months (short-term survivors, *p* = 0.021, **Figure 2D**). Short-term survival was associated with *Bacteroides, Prevotella, Fusobacterium, Selenomonas, Fretibacterium*, and *Porphyromonas*. In contrast, *Sphingomonas, Corynebacterium, Curvibacter, Enhydrobacter, Paracoccus, Rothia, Caulobacter*, and *Dermacoccus* were associated with long-term survival (**Figure 3**). The mean relative abundances of *Bacteroides, Selenomonas, Fretibacterium*, and *Peptostreptococcus* were higher in short-term survivors, while *Rothia, Curvibacter, Corynebacterium*, and *Sphingomonas* were more abundant in long-term survivors (**Table 2**).

**Table 2:** Average abundancies of microbiome genus between short term and long-term survival.

Genus	Long term survivor (≥ 24 months, n = 49)	Short term survivor (<24 months, n = 71)	*p* value
*Prevotella*	0.011	0.035	0.052
*Bacteroides*	0.018	0.041	0.040
*Fusobacterium*	0.014	0.013	0.923
*Selenomonas*	0	0.006	0.023
*Fretibacterium*	0	0.004	0.039
*Porphyromonas*	0	0.002	0.016
*Dermacoccus*	0.001	0	0.099
*Caulobacter*	0.001	0	0.101
*Rothia*	0.002	0	0.047
*Paracoccus*	0.016	0	0.054
*Enhydrobacter*	0.003	0.001	0.199
*Curvibacter*	0.010	0.005	0.049
*Corynebacterium*	0.016	0.006	0.024
*Sphingomonas*	0.015	0.004	0.038
*Peptostreptococcus*	0	0.004	0.038
*Leptotrichia*	0	0.004	0.060

### 3.5 Prognostic implications of cancer tissue microbiome

**Figure 4:**
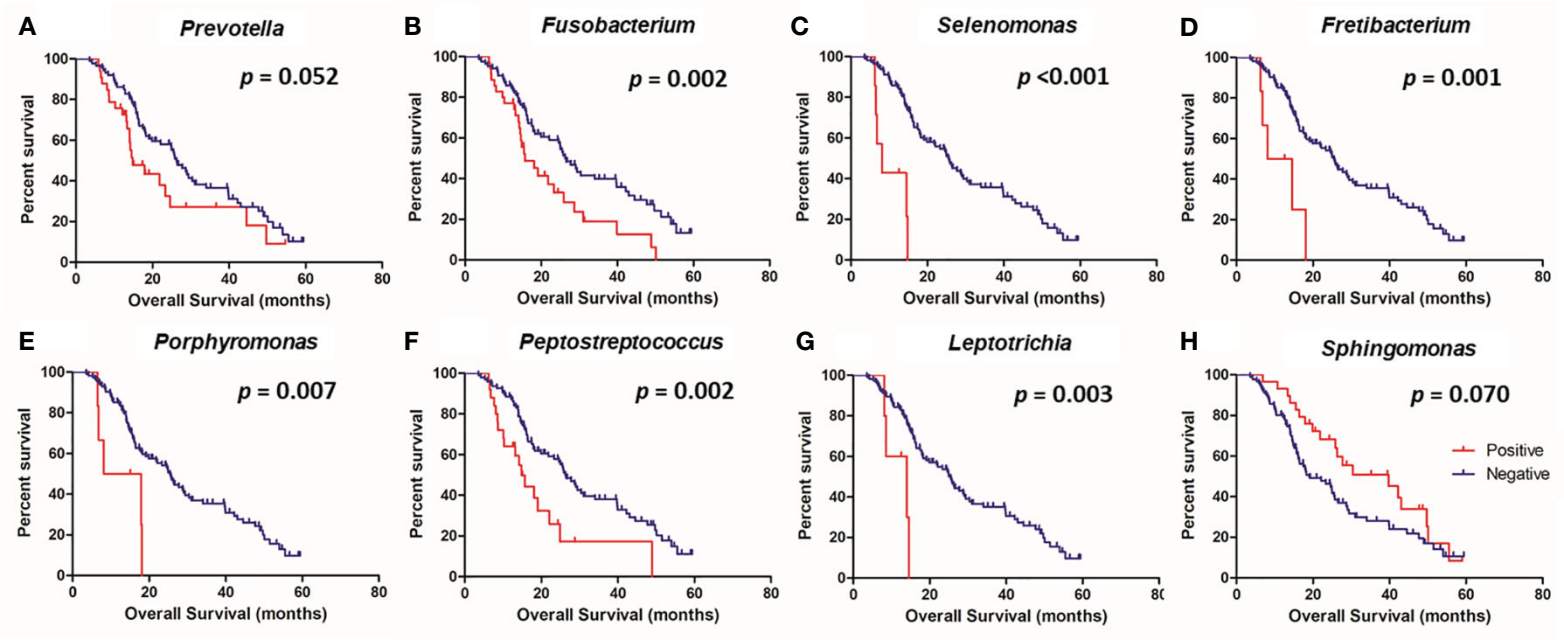
Kaplan-Meier analysis of overall survival according to the presence or absence of specific microbes in the colorectal tissue. *Prevotella* **(A)**, *Fusobacterium* **(B)**, *Selenomonas* **(C)**, *Fretibacterium* **(D)**, *Porphyromonas* **(E)**, *Peptostreptococcus* **(F)**, *Leptotrichia* **(G)**, and *Sphingomonas* **(H)**.

The survival analysis revealed that the presence of *Prevotella* (*p* = 0.050), *Fusobacterium* (*p* = 0.002), *Selenomonas* (*p <*0.001), *Fretibacterium* (*p* = 0.001), *Porphyromonas* (*p* = 0.007), *Peptostreptococcus* (*p* = 0.002), and *Leptotrichia* (*p* = 0.003) were associated with poor OS, while the presence of S*phingomonas* was associated with good OS (*p* = 0.070) (**Figure 4**). In a multivariate analysis, the presence of *Selenomonas* (HR, 6.35; 95% CI, 2.38–16.97; *p <*0.001) remained a significant poor prognostic factor, along with high serum CEA level (**Table 3**).

**Table 3:** Variables associated with overall survival.

	Univariate	Multivariate	
	*P* value	HR (95% CI)	*P* value
Age (≥ 60)	0.083	1.44 (0.87–2.40)	0.158
Male	0.975		
ECOG ≥ 2	0.034	1.16 (0.69–1.93)	0.581
Primary site	0.517		
Presence of RAS mutation	0.129		
No. of metastatic sites (≥ 3)	0.022	1.58 (0.90–2.78)	0.109
CEA ≥ 10 ng/mL	<0.001	2.22(1.37–3.59)	0.001
Presence of each microbiome			
* Bacteroides*	0.154		
* Prevotella*	0.052	1.13 (0.63–2.04)	0.681
* Fusobacterium*	0.002	0.68 (0.35–1.32)	0.259
* Selenomonas*	<0.001	6.35 (2.38–16.97)	<0.001
* Fretibacterium*	0.002	0.40 (0.09–1.84)	0.240
* Porphyromonas*	0.011	1.68 (0.58–4.89)	0.339
* Peptostreptococcus*	0.003	1.49 (0.79–2.82)	0.221
* Leptotrichia*	0.006	0.99 (0.16–6.33)	0.995
* Sphingomonas*	0.070	0.80 (0.44–1.47)	0.477
* Corynebacterium*	0.263		
* Curvibacter*	0.118		
* Enhydrobacter*	0.319		
* Paracoccus*	0.230		
* Rothia*	0.198		
* Caulobacter*	0.491		
* Dermacoccus*	0.705		

HR, hazard ratio; CI, confidence interval; ECOG, Eastern Cooperative Oncology Group; CEA, carcinoembryonic antigen.

## 4 Discussion

We characterized the composition of the tissue microbiome in patients with metastatic CRC and identified potential microbiome genera that may contribute to the prediction of survival outcomes in this population. Some clinical parameters are associated with microbiome diversity. The rectal origin showed higher microbiome richness and distinct beta diversity, in accordance with previous reports (29, 30). Patients with high CEA levels showed higher microbiome richness, but lower diversity. Peritoneal invasion (cT4) was associated with lower diversity. These are clinical parameters of poor prognosis in CRC; however, we did not find any significant differences in alpha diversity according to the clinical outcomes. Microbiome feature was associated with survival outcomes. The presence of *Prevotella, Fusobacterium, Selenomonas, Fretibacterium, Porphyromonas, Peptostreptococcus* and *Leptotrichia* was associated with shorter OS, while *Sphingomonas* was associated with longer OS.

One strength of our study is that we performed microbiome analysis using tissue samples from patients with metastatic CRC instead of stool samples. The colon tissue microbiome directly reflects features of the TME, and thus is less affected by extrinsic factors such as diet, drug exposure, gut preparation, or sample storage processes than stool samples (31). Studies comparing local tumor tissues with stool microbiome patterns have yielded discordant results (11, 29, 30). Thus, direct determination of the tissue-associated microbiome from primary CRC tumors may prove useful in future studies to confirm its prognostic implications.

Interestingly, the microbiomes related to poor prognosis in our study belonged to the oral cavity. Microbiomes living in the oral cavity can translocate to the lower gut and participate in pathogenesis (32). Significant enrichment of orally originated microbiomes has been observed in CRC tissues compared to normal tissues (12, 16, 29, 33). *Fusobacteria*, particularly *F. nucleatum*, is an established part of the oral microbiome and has the potential to contribute to CRC carcinogenesis. Its presence in CRC is associated with prognosis after surgical resection (8, 10, 17). These bacteria induce EMT, dysregulate the E-cadherin/β-catenin complex, and secrete matrix metalloproteinases or chemokines, which enhance tumor aggressiveness and metastasis (34–37). Few studies reported a prognostic role of *F. nucleatum* in the metastatic setting (38, 39). In the present study, *Fusobacterium* was associated with poor survival outcomes. At the species level, *F. nucleatum* was detected in 15 (12.5%) patients, and its presence was significantly associated with shorter OS (*p <*0.001; data not shown).

The prognostic significance of other bacteria in CRC has not been widely studied (16). We also identified other bacterial genera that may be contributing factors. For example, *Porphyromonas* promotes cell proliferation or inflammatory responses via interaction with TLR2 or TLR4 in preclinical models, and is associated with the prognosis of lung or esophageal cancer (40, 41). *Peptostreptococcus* involved in CRC carcinogenesis via direct interaction with integrin α2/β1 activates PI3K-ALK-NF-ĸB signaling and immune modulation (42). *Leptotrichia* and *Selenomonas* are associated with high-grade CRC; however, their prognostic significance has not yet been reported (30). We also found that some prognostic parameters correlated with each microbiome. *Fusobacterium* was detected more frequently in patients with high CEA level (*p <*0.001) and high tumor burden (*p* = 0.009). Patients with a high tumor burden showed more frequent *Leptotrichia* (*p* = 0.022) and *Fretibacterium* (*p* = 0.048). In this study, *Sphingomonas* was associated with favorable prognosis, which has been reported in breast, lung, and gastric cancer (43–45).

Most patients with CRC received 5-FU based chemotherapy. *F. nucleatum* may cause 5-FU chemoresistance via mechanisms involving aberrant EMT, autophagy, apoptosis, or tumor cell cycle regulation in CRC (26, 34, 46). *Porphyromonas* enhances the viability of cancer cells exposed to chemotherapeutic agents, including 5-FU, by modifying apoptosis and cell cycle signaling in esophageal cancer (40). Several studies have reported that short-chain fatty acids (SCFA), including those produced by butyrate-producing bacteria, may be associated with 5-FU chemo-sensitivity in CRC. Yuan et al. reported that antibiotic administration decreased 5-FU efficacy and was associated with a decrease of SCFA-producing bacteria, such as *Blautia, Roseburia, Anaerotruncus, Oscillibacter*, and *Bacteroidetes*, which could induce inflammation and abrogate antitumor activity. Another study reported that *Roseburia, Dorea*, and *Anaerostipes* in stool samples of patients with locally advanced rectal cancer were overrepresented in 5-FU chemoradiation responders (47). In the present study, these microbiomes were not associated with 5-FU efficacy or clinical outcomes. *Porphyromonas* or *Prevotella*, which might belong to the butyrate-producing microbiome (48–50), are associated with poor prognosis.

Our study has some limitations. First, it was conducted at a single institution, and the sample size was relatively small. Due to its retrospective nature, some important information, including prior antibiotic or probiotic exposure, was missing. Second, we used FFPE samples, which may present sequencing quality or contamination issues during processing. To minimize these issues, we filtered out reads of low quality, and ASVs with ambiguous genera annotations or those found in less than 1% of the samples were also discarded. Some microbiome was found in a small number of patients, so results should be interpreted in caution. Third, we investigated genera instead of species because of the low sensitivity of the 16S rRNA sequencing method for classifying species (51). Shotgun metagenomic sequencing was not performed because of high costs and excessive contamination with human DNA in the tissue samples (3, 4). Some studies have suggested that finer taxonomic resolution could impede the clinical availability of microbiome analyses for CRC (37).

In conclusion, we performed comprehensive tumor microbiome sequencing in patients with metastatic CRC and identified distinct tumor microbiome features associated with survival outcomes in this population. The presence of *Selenomonas*, along with the typical oral carcinogenic microbiome in CRC tissues, may be a useful prognostic biomarker. To confirm their clinical value, these microbiomes should be validated from RNA-ISH method and in stool samples from a large cohort.

## Data availability statement

The data presented in the study are deposited in the NIH's Short Read Archive repository, accession number PRJNA808420: https://www.ncbi.nlm.nih.gov/sra/PRJNA808420.

## Ethics statement

The studies involving humans were approved by the institutional review boards (IRB) of St. Vincent’s Hospital (No. VC19TESI0114) and Stanford Hospital (IRB number 55131). The studies were conducted in accordance with the local legislation and institutional requirements. The participants provided their written informed consent to participate in this study.

## Author contributions

HA: Conceptualization, Data curation, Formal analysis, Funding acquisition, Investigation, Methodology, Resources, Writing – original draft. MP: Formal analysis, Writing – original draft. HL: Formal analysis, Software, Supervision, Validation, Writing – review & editing. BL: Data curation, Methodology, Supervision, Writing – review & editing. DP: Formal analysis, Writing – review & editing. AA: Project administration, Resources, Writing – review & editing. AH: Data curation, Methodology, Supervision, Writing – review & editing. GS: Data curation, Methodology, Supervision, Writing – review & editing. HJ: Conceptualization, Funding acquisition, Project administration, Resources, Supervision, Writing – review & editing.
