# Supplementary material for: Tumor-associated microbiome features of metastatic colorectal cancer and clinical implications
Source: Front Oncol. 2024 Jan 18;13:1310054. doi: 10.3389/fonc.2023.1310054 (PMC10833227; doi:10.3389/fonc.2023.1310054)
Supplement: Supplementary file 1 [file DataSheet_1.docx]

**Supplementary Figures**


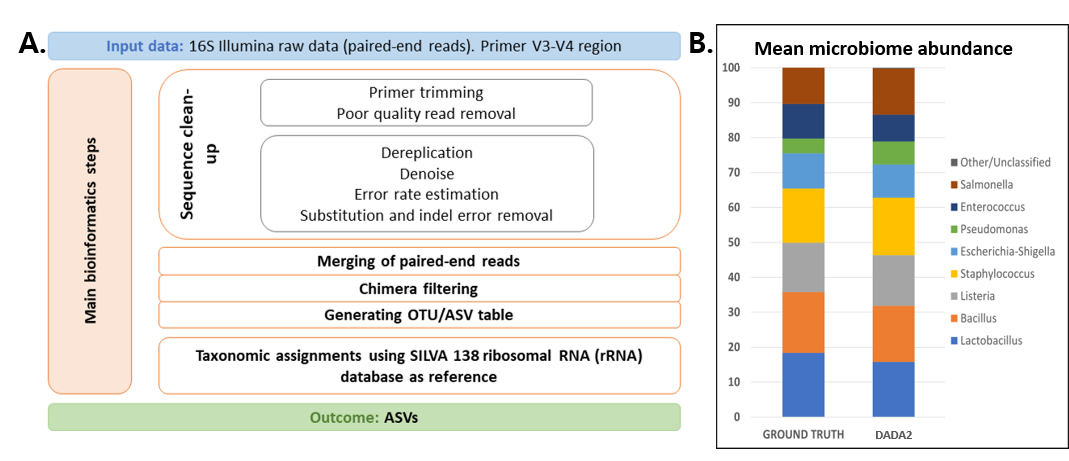


**Supplementary figure 1.** Overview of the workflows used by DADA2 (A), and mean microbiome abundance in a positive control (B). OTU, operational taxonomic units; ASV, amplicon sequence variant

**
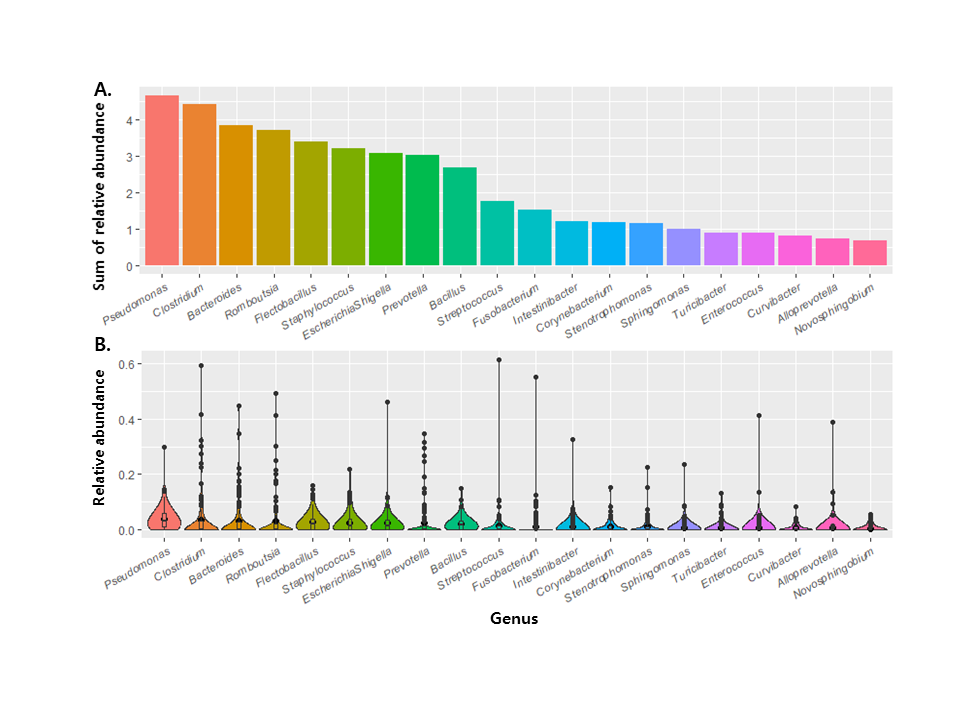
 Supplementary figure 2.** Sum of relative abundance (A), and relative abundance (B) of genus
